# Association of Dementia Risk With Focal Epilepsy and Modifiable Cardiovascular Risk Factors

**DOI:** 10.1001/jamaneurol.2023.0339

**Published:** 2023-03-27

**Authors:** Xin You Tai, Emma Torzillo, Donald M. Lyall, Sanjay Manohar, Masud Husain, Arjune Sen

**Affiliations:** 1 Nuffield Department of Clinical Neurosciences, University of Oxford, Oxford, United Kingdom; 2 Division of Clinical Neurology, John Radcliffe Hospital, Oxford University Hospitals Trust, Oxford, United Kingdom; 3 Epilepsy Department, National Hospital for Neurology and Neurosurgery, University College London, London, United Kingdom; 4 Institute of Health and Wellbeing, University of Glasgow, Glasgow, United Kingdom; 5 Department of Experimental Psychology, University of Oxford, Oxford, United Kingdom; 6 Oxford Epilepsy Research Group, NIHR Biomedical Research Centre, Nuffield Department of Clinical Neurosciences, John Radcliffe Hospital, Oxford, United Kingdom

## Abstract

**Importance:**

Epilepsy has been associated with cognitive impairment and potentially dementia in older individuals. However, the extent to which epilepsy may increase dementia risk, how this compares with other neurological conditions, and how modifiable cardiovascular risk factors may affect this risk remain unclear.

**Objective:**

To compare the differential risks of subsequent dementia for focal epilepsy compared with stroke and migraine as well as healthy controls, stratified by cardiovascular risk.

**Design, Setting, and Participants:**

This cross-sectional study is based on data from the UK Biobank, a population-based cohort of more than 500 000 participants aged 38 to 72 years who underwent physiological measurements and cognitive testing and provided biological samples at 1 of 22 centers across the United Kingdom. Participants were eligible for this study if they were without dementia at baseline and had clinical data pertaining to a history of focal epilepsy, stroke, or migraine. The baseline assessment was performed from 2006 to 2010, and participants were followed up until 2021.

**Exposures:**

Mutually exclusive groups of participants with epilepsy, stroke, and migraine at baseline assessment and controls (who had none of these conditions). Individuals were divided into low, moderate, or high cardiovascular risk groups based on factors that included waist to hip ratio, history of hypertension, hypercholesterolemia, diabetes, and smoking pack-years.

**Main Outcomes and Measures:**

Incident all-cause dementia; measures of executive function; and brain total hippocampal, gray matter, and white matter hyperintensity volumes.

**Results:**

Of 495 149 participants (225 481 [45.5%] men; mean [SD] age, 57.5 [8.1] years), 3864 had a diagnosis of focal epilepsy only, 6397 had a history of stroke only, and 14 518 had migraine only. Executive function was comparable between participants with epilepsy and stroke and worse than the control and migraine group. Focal epilepsy was associated with a higher risk of developing dementia (hazard ratio [HR], 4.02; 95% CI, 3.45 to 4.68; *P* < .001), compared with stroke (HR, 2.56; 95% CI, 2.28 to 2.87; *P* < .001), or migraine (HR, 1.02; 95% CI, 0.85 to 1.21; *P* = .94). Participants with focal epilepsy and high cardiovascular risk were more than 13 times more likely to develop dementia (HR, 13.66; 95% CI, 10.61 to 17.60; *P* < .001) compared with controls with low cardiovascular risk. The imaging subsample included 42 353 participants. Focal epilepsy was associated with lower hippocampal volume (mean difference, −0.17; 95% CI, −0.02 to −0.32; *t* = −2.18; *P* = .03) and lower total gray matter volume (mean difference, −0.33; 95% CI, −0.18 to −0.48; *t* = −4.29; *P* < .001) compared with controls. There was no significant difference in white matter hyperintensity volume (mean difference, 0.10; 95% CI, −0.07 to 0.26; *t* = 1.14; *P* = .26).

**Conclusions and Relevance:**

In this study, focal epilepsy was associated with a significant risk of developing dementia, to a greater extent than stroke, which was magnified substantially in individuals with high cardiovascular risk. Further findings suggest that targeting modifiable cardiovascular risk factors may be an effective intervention to reduce dementia risk in individuals with epilepsy.

## Key Points

**Question:**

To what extent does having focal epilepsy compared with stroke or migraine increase the risk of developing dementia, and how is it affected by modifiable cardiovascular risk factors?

**Findings:**

This cross-sectional study of 495 149 participants aged 38 to 72 years without dementia at baseline demonstrated that participants with epilepsy and high cardiovascular risk were more than 13 times more likely to develop dementia compared with control participants with low cardiovascular risk while participants with stroke and high cardiovascular risk were almost 6 times more likely to develop dementia. Having epilepsy was associated with higher incident dementia risk than a history of stroke.

**Meaning:**

This study found that epilepsy was associated with a significant risk of developing dementia, which was magnified substantially by cardiovascular risk.

## Teaser

This cross-sectional study examines data from the UK Biobank to explore the association between focal epilepsy compared with stroke or migraine and the risk of developing dementia and how that risk is affected by modifiable cardiovascular risk factors.

## Introduction

Epilepsy is a common neurological condition characterized by unprovoked seizures. The incidence of epilepsy is highest in older life and progressively increases after 55 years of age.^1,2,3^ As individuals with epilepsy age, studies suggest an increased risk of cognitive impairment and potentially dementia.^4,5^ However, the extent to which epilepsy affects dementia risk and potential underlying mechanisms remains unclear. In addition, there are no specific clinical guidelines around mitigating the risk of dementia in epilepsy.

While recent evidence has highlighted a shared pathology link of tau accumulation in epilepsy and dementia,^6,7,8^ another intriguing line of inquiry is the role of modifiable cardiovascular risk factors that contribute to dementia risk in the general aging population.^9,10^ Established stroke is a risk factor for developing epilepsy in older adults^11^; however, the effect of upstream cardiovascular risk factors is less clear with conflicting findings.^12,13^ Similarly, while poststroke epilepsy is considered predictive of cognitive outcomes,^14^ how dementia risk in epilepsy may change according to an individual’s burden of modifiable cardiovascular risk factors in the absence of stroke remains unknown.

A recent systematic review^5^ identified disease duration, seizure frequency, and antiseizure medication use as potential predictors of cognitive impairment in epilepsy, although studies were limited by small samples of patients and controls and the lack of consideration for lifestyle and cardiovascular risk factors.^15,16,17^ Correspondingly, a meta-analysis examining dementia risk in epilepsy identified similar limitations and was unable to calculate period prevalence owing to insufficient pooled sample size.^3^ To guide clinical management, it is important to understand the epilepsy-related dementia risk compared with other neurological conditions to provide comparator context for clinical decisions.

In this study, we analyzed the risk associated with developing dementia across a range of neurological conditions in the UK Biobank prospective cohort. Specifically, our aim was to determine the extent to which focal-onset epilepsy is associated with risk of developing dementia compared with individuals with stroke or migraine, 2 other nondegenerative neurological conditions, as well as healthy controls. We hypothesized that epilepsy would be associated with higher dementia incidence than migraine and controls but less than stroke, which is strongly linked to vascular cognitive impairment and dementia. Further, we determined the extent to which having low cardiovascular risk is associated with reduced risk of dementia in epilepsy as this may help develop dementia risk reduction strategies for people with epilepsy.

## Methods

This cross-sectional study is based on data from the UK Biobank, a population-based cohort of more than 500 000 participants aged 38 to 72 years who underwent physiological measurements and cognitive testing and provided biological samples at 1 of 22 centers across the United Kingdom between 2006 and 2010.^18^ A subset of participants reattended for brain imaging between 2014 and 2020.^19^ All participants provided written informed consent. UK Biobank received approval from the North West Multicenter Research Ethics Committee. This study followed the Strengthening the Reporting of Observational Studies in Epidemiology (STROBE) reporting guideline.

The primary study objective was to investigate the risk of incident dementia associated with having focal epilepsy compared with stroke or migraine and healthy controls at baseline study assessment. Participants with prevalent dementia at baseline assessment (<1%) were excluded as were those with other neurological conditions, including a history of central nervous system infection, encephalitis, meningitis, amyotrophic lateral sclerosis, multiple sclerosis, Parkinson disease, or previous subdural or subarachnoid hemorrhage. Baseline diagnoses were identified using self-report and hospital inpatient records (UK Biobank codes are found in eTable 1 in Supplement 1).

### Dementia and Focal Epilepsy Diagnoses

All-cause dementia cases were identified during longitudinal follow-up from hospital inpatient records using codes from the *International Classification of Diseases, Ninth Revision* (*ICD-9*), and *International Statistical Classification of Diseases and Related Health Problems, Tenth Revision* (*ICD-10*), for Alzheimer disease and other dementia classifications or from death register linkage data as an underlying or contributory cause.

The epilepsy subgroup was restricted to focal-onset, nongenetic epilepsy at baseline based on *ICD* codes. We excluded individuals coded for a genetically associated epilepsy, including “generalized idiopathic epilepsy and epileptic syndromes” (G40.3), such as syndromes of juvenile myoclonic epilepsy and childhood absence epilepsies, and “other generalized epilepsy and epileptic syndromes” (G40.4), which includes epileptic encephalopathies such as Lennox-Gaustaut and West syndrome, because these can be associated with clear cognitive deficits related to an underlying genetic mechanism or developmental delay.^20^ To investigate the potential confounding effects of antiseizure medication, we compared the association between cognitive scores and number of antiseizure medications (list in eTable 2 in Supplement 1) for individuals with a diagnosis of focal epilepsy and those without a diagnosis but taking the medication for another reason. Information on seizure onset zone in the brain was not available; however, the majority of focal-onset, acquired epilepsy may be of temporal lobe origin.^21^

### Cardiovascular Risk Score

Cardiovascular risk was assessed based on a previously published score^22^ with a point given for a diagnosis or being treated for hypertension, high cholesterol, or diabetes (1 point for each condition), waist to hip ratio (1 point if greater than the sex-specific threshold set by World Health Organization guidelines),^23^ smoking pack-years (1 point if more than 20 pack-years), and *APOE* e4 allele status (2 points for 2 e4 alleles, 1 for a single e4 allele, and 0 for any other allele combination or when allele status was unclear). Participants were placed in low- (a score of 0), moderate- (1 and 2), or high-risk (≥3) groups based on quintile boundaries 1, 2 to 4, and 5, respectively.

### Cognitive Testing

UK Biobank cognitive testing was computer-based and performed at the initial baseline visit, repeated at the imaging visit, and taken via online questionnaire. Not all cognitive tasks were performed at each instance while some were repeated. We analyzed data from 5 tasks of working memory or speed of processing and used the first available time point data. This included a pairs-matching and snap reaction time, trail-making, tower-rearranging, and symbol-digit substitution tasks and has been described in detail elsewhere.^24^ Reliability and retest effects over time for these cognitive tasks have been previously assessed.^25^

### Main Covariates

All full models were adjusted for age (continuous), sex (female vs male), education (categorized as higher [college or university degree or other professional qualification], upper secondary [second or final stage of secondary education], lower secondary [first stage of secondary education], vocational [work-related qualifications], or other), socioeconomic status (categories derived from Townsend deprivation index^26^ quintiles 1, 2 to 4, and 5), and cardiovascular risk group.

### Brain Imaging Variables

Magnetic resonance imaging (MRI) data were acquired on a Skyra 3-T scanner (Siemens), including high-resolution, T1-weighted, 3-dimensional magnetization-prepared gradient echo structural images and T2-weighted fluid-attenuated inversion recovery images. Full imaging protocols and processing pipeline have been previously described.^27^ We used imaging summary statistics of total hippocampal, gray matter, and white matter hyperintensity volumes. These regions were chosen because previous epilepsy studies have found hippocampal atrophy^28,29,30^ and hippocampal tau deposition.^6^ Total gray matter volume is a useful measure of widespread, global change while white matter hyperintensity is a useful marker of vascular burden. Median absolute deviation was used to exclude outliers, and volumes were adjusted for potentially confounding baseline measures of age, age squared, head size, and imaging site.^27^

### Statistical Analysis

Confirmatory factor analysis (CFA) was performed on cognitive variables to produce a continuous, summary latent measure of working memory and reaction time, which we termed *executive function* for simplicity (this method has been previously described).^22,24^ In brief, cognitive variables were preprocessed to correct for heavily skewed distribution prior to CFA. Standard fit indices were measured with higher comparative fit index and Tucker-Lewis index considered better (>0.9 are commonly used as acceptable fit cutoffs) while lower root mean square error of approximation and standardized root mean square error residual are considered better (<0.06 and <0.08, respectively, are commonly used cutoffs for acceptable fit). Estimating a latent variable has the methodological advantage of controlling measurement error that can artificially reduce the relationship between measured variables in standard univariate analyses.^31^ Missing cognitive data were estimated using full information maximum likelihood, which gives unbiased parameter estimates and standard errors.

We examined mutually exclusive groups of focal epilepsy, stroke, and migraine. Combinations of conditions, such as having epilepsy and stroke, were not reported because of the small sample sizes. The association between executive function across age was examined for each group. We then controlled for age in 2 different ways for robustness: using a model-free, sliding window approach with fixed age-quantile widths moved along the age distribution (described previously^24,32,33^) or by including age as a covariate in a general linear model along with other baseline characteristics. We compared the difference in executive function between condition groups using analysis of variance with post hoc Tukey analysis to account for pairwise or multiple comparisons. *P* values were 2-sided with statistical significance set at *P* < .05 for all analyses. Brain measures were analyzed in the epilepsy subgroup using the same methods.

Hazard ratios (HRs) were calculated using Cox proportional hazards regression models with time to incident all-cause dementia as the dependent variable. We calculated HRs for mutually exclusive groups of focal epilepsy, stroke, and migraine compared with none of these conditions as the baseline. For our main model, we tested the dementia risk associated with having focal epilepsy, stroke, or neither condition, stratified by cardiovascular risk groups (9 categories with low cardiovascular risk and neither epilepsy or stroke as the baseline). The migraine subgroup had similar HRs to those for the control group from initial testing and was not included in this model. For the main exposures and covariates, there were less than 3% missing or not known data, and complete case analysis was applied. Participants were considered at risk for dementia from baseline until the date of first diagnosis, death, loss to follow-up, or last surveyed hospital admission date (March 31, 2021, for England and Scotland and February 28, 2018, for Wales), whichever came first. These censoring dates were recommended by UK Biobank as the data were estimated to be more than 90% complete in England, Scotland, and Wales.

Secondary data analyses examined dementia risk with different follow-up durations of 10 years and 5 to 14 years to consider earlier risk of developing dementia and potential reverse causality, respectively. Further sensitivity analysis considered dementia risk associated with stroke and epilepsy stratified by a cardiovascular risk score that did not include *APOE* e4 genetic information to consider only modifiable lifestyle risk. The association between dementia risk and epilepsy controlling for number of antiseizure medications was investigated within the epilepsy subgroup only. Analyses were done in MATLAB R2018a or in R version 4.0.3 using the lavaan^34^ or survival package.

## Results

The UK Biobank cohort comprised 502 536 participants at baseline. After excluding those who did not meet the inclusion criteria (n = 7216) and those with prevalent dementia at baseline (n = 120), our study included 495 149 individuals (eFigure 1 in Supplement 1). Participants had a mean (SD) age of 57.5 (8.1) years, and 250 752 (54.5%) were female (Table). Over 5 803 006 total follow-up years (median, 12.0 years; IQR, 11.2-12.7), 6115 cases of all-cause incident dementia were observed.

**Table.** Baseline Characteristics of Study Participants

Characteristic	No. (%)^a^							
	Control group		Epilepsy		Stroke		Migraine	
	No incident dementia	Incident dementia	No incident dementia	Incident dementia	No incident dementia	Incident dementia	No incident dementia	Incident dementia
No. of participants	464 138	5471	4121	198	6679	337	15 835	174
Age, mean (SD), y	57.4 (8.1)	65.3 (4.8)	57.1 (8.1)	63.5 (6.1)	61.5 (6.9)	65.4 (4.7)	56.1 (7.8)	63.7 (6.2)
Sex								
Female	250 752 (54.0)	2619 (47.9)	2071 (50.3)	77 (38.9)	2766 (41.4)	119 (35.3)	12 363 (78.1)	117 (67.2)
Male	213 386 (46.0)	2852 (52.1)	2050 (49.7)	121 (61.1)	3913 (58.6)	218 (64.7)	3472 (21.9)	57 (32.8)
Education^b^								
Higher	217 148 (46.8)	1813 (33.1)	1623 (39.4)	55 (27.8)	2257 (33.8)	86 (25.5)	7810 (49.3)	53 (30.4)
Upper secondary	58 464 (12.6)	550 (10.1)	547 (13.3)	23 (11.6)	807 (12.1)	33 (9.8)	1939 (12.2)	17 (9.8)
Lower secondary	25 227 (5.4)	253 (4.6)	202 (4.9)	10 (5.1)	302 (4.5)	19 (5.6)	919 (5.8)	11 (6.3)
Vocational	77 047 (16.6)	746 (13.6)	708 (17.1)	23 (11.6)	1045 (15.6)	42 (12.5)	2793 (17.6)	22 (12.6)
Other	86 252 (18.6)	2109 (38.5)	1047 (25.4)	85 (42.9)	2268 (34.0)	157 (46.6)	2374 (15.0)	71 (40.8)
Socioeconomic status quintile^c^								
1 (Least deprived)	93 326 (20.1)	990 (18.1)	641 (15.5)	28 (14.1)	959 (14.4)	39 (11.6)	3161 (20.0)	31 (17.8)
2-4	278 742 (60.1)	3151 (57.6)	2317 (56.2)	96 (48.5)	3629 (54.3)	176 (52.2)	9588 (60.5)	91 (52.3)
5 (Most deprived)	91 495 (19.7)	1323 (24.2)	1166 (28.2)	74 (37.4)	2084 (31.2)	122 (36.2)	3061 (19.3)	52 (29.9)
NA	575 (0.1)	7 (0.1)	2 (0.05)	0	7 (0.1)	0	25 (0.2)	0
Cardiovascular risk group (score)^d^								
Low (0)	127 055 (27.3)	495 (9.0)	997 (24.2)	21 (10.6)	531 (8.0)	13 (3.9)	5490 (34.7)	31 (17.8)
Moderate (1-2)	258 527 (55.7)	2695 (49.3)	2309 (56.0)	91 (46.0)	2700 (40.4)	97 (28.8)	8585 (54.2)	87 (50.0)
High (≥3)	78 556 (16.9)	2281 (41.7)	815 (19.8)	86 (43.4)	3448 (51.6)	227 (67.4)	1760 (11.1)	56 (32.2)

Abbreviation: NA, not available.

^a^ Percentages may not sum to 100 because of rounding.

^b^ Higher education defined as college/university degree or other professional qualification; upper secondary, second/final stage of secondary education; lower secondary, first stage of secondary education; vocational, work-related practical qualifications.

^c^ Socioeconomic status assessed on the Townsend deprivation index, which combines information on social class, employment, car availability, and housing.

^d^ Cardiovascular risk group was calculated from a cardiovascular risk score with points given for history of hypertension, high cholesterol, diabetes, waist to hip ratio, smoking history, and genetic *APOE* genotype.

At baseline assessment, 3864 participants (0.78%) had a diagnosis of focal epilepsy only, 6397 (1.3%) had a history of stroke only, and 14 518 (2.93%) had migraine only. There were 134 249 (27.1%) participants with low cardiovascular risk while 274 098 (55.4%) and 86 802 (17.5%) had a moderate and high cardiovascular risk, respectively (eTable 3 in Supplement 1).

### Executive Function Between Patient Groups and Controls

**Figure 1.**
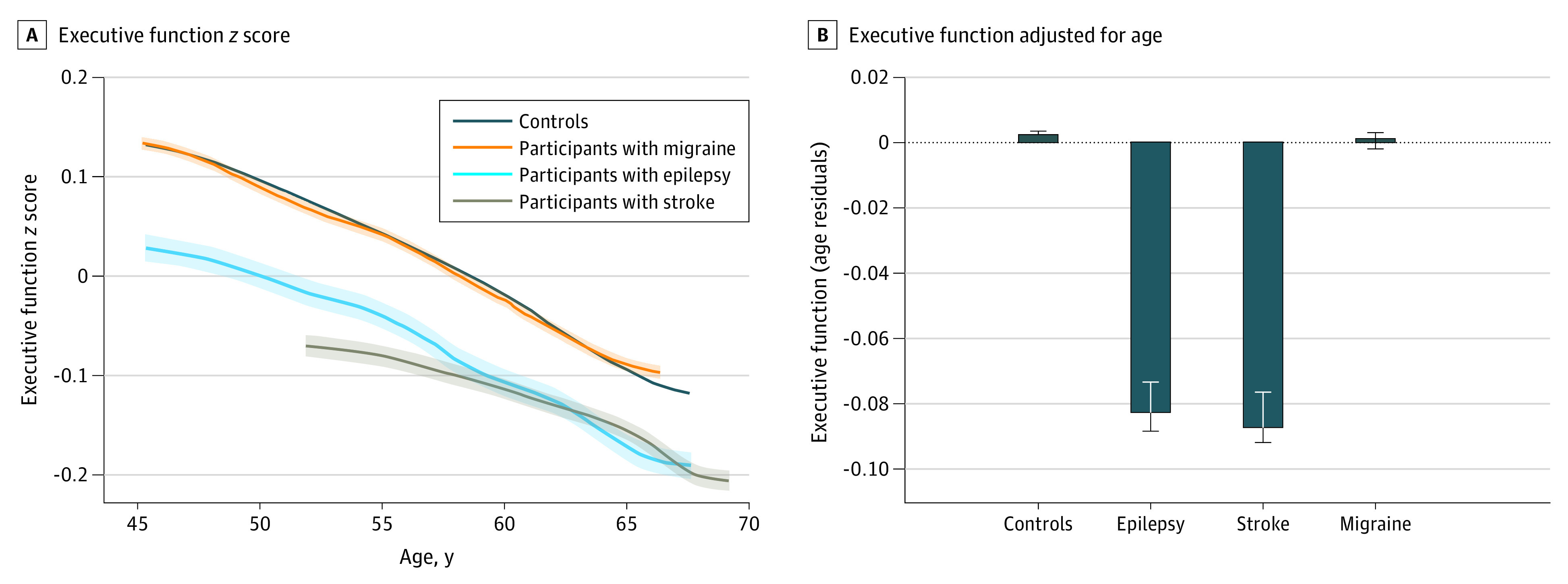
Association Between Executive Function and Age in Participants With Epilepsy, Stroke, or Migraine and Control Participants Participants with epilepsy and stroke had lower executive function (*z*-scored values) across all ages compared with controls with no history of epilepsy, stroke, or migraine (A). The shaded areas represent standard error. When adjusting for age (B) using an age-residual approach, there was a group difference (*F*_3,489 069_ = 201.97, *P* < .001). Post hoc pairwise comparison showed that having epilepsy or stroke was associated with significantly lower executive function compared with controls (*t* = 14.70, *P* < .001, and *t* = 19.90, *P* < .001, respectively, using post hoc Tukey analysis) and participants with migraine (*t* = −12.82, *P* < .001, and *t* = −16.35, *P* < .001, respectively). There was no significant difference in executive function between participants with migraine and controls (Tukey analysis *P* = .91) or between participants with epilepsy or stroke (Tukey analysis *P* = .91). All groupings shown were mutually exclusive; ie, the epilepsy group had no history of stroke or migraine.

A continuous cognitive function latent variable of executive function was estimated from 5 cognitive tasks of working memory or speed of processing (model and fit indices are shown in eFigure 2 in Supplement 1). Executive function declined uniformly with age among all groups, consistent with previous findings (Figure 1).^22,24^ We controlled for age using a model-free residual method and showed that focal epilepsy was associated with lower executive function than controls and migraine (mean difference, −0.09; 95% CI, −0.07 to −0.10; *t* = 14.70; *P* < .001, and mean difference, −0.08; 95% CI, −0.10 to −0.10; *t* = −12.82, *P* < .001, respectively, using post hoc Tukey analysis). There was no significant difference between executive function in focal epilepsy or stroke (mean difference, 0.01; 95% CI, −0.01 to 0.02; *t* = 14.70; post hoc Tukey *P* = .91) (eTable 4 in Supplement 1). The associations between executive function and neurological conditions remained consistent with a fully adjusted linear model accounting for other baseline characteristics (eTable 5 in Supplement 1).

### Dementia Outcomes

**Figure 2.**
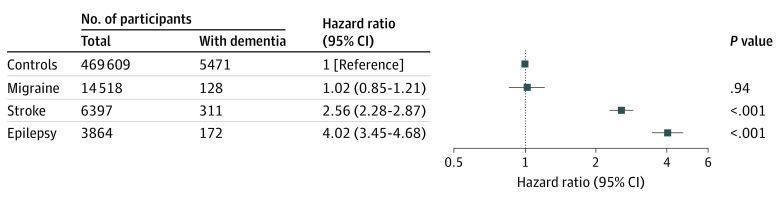
Risk of Incident Dementia by Neurological Disease Status at Baseline The control group had no migraine, stroke, or epilepsy. The model was adjusted for age, sex, education, socioeconomic status, and assessment center.

**Figure 3.**
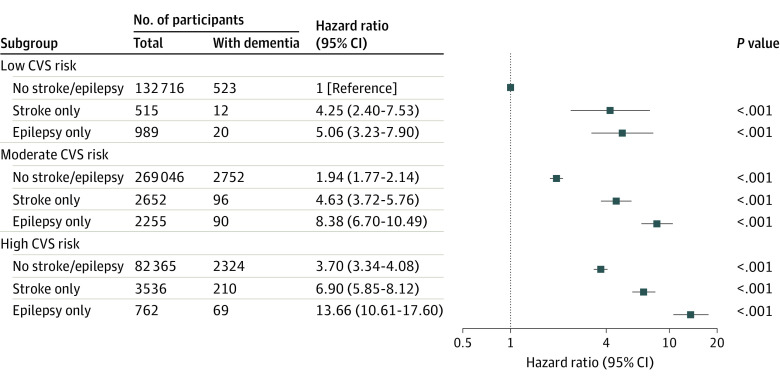
Risk of Incident Dementia Associated With Focal Epilepsy and Stroke According to Cardiovascular (CVS) Risk The model was adjusted for age, sex, education, socioeconomic status, and assessment center.

During the study follow-up, the adjusted HR for incident dementia was 4.02 (95% CI, 3.45-4.68) for participants with focal epilepsy (Figure 2), which was higher than stroke (HR, 2.56; 95% CI, 2.28-2.87) and migraine (HR, 1.02; 95% CI, 0.85-1.21). Within moderate and high cardiovascular risk groups, having focal epilepsy was associated with a higher risk of developing dementia compared with stroke (Figure 3). Of participants with high cardiovascular risk and focal epilepsy, 69 of 762 (9.1%) developed dementia compared with 523 of 132 716 (0.4%) control participants with low cardiovascular risk (HR, 13.66; 95% CI, 10.61-17.60). When considering only individuals with high cardiovascular risk, those with focal epilepsy had an HR of 3.73 (95% CI, 2.94-4.76) for developing dementia compared with controls while participants with stroke had an HR of 1.90 (95% CI, 1.65-2.20) compared with controls. Risks of dementia within moderate and low cardiovascular risk groups are detailed in eTable 6 in Supplement 1).

### Sensitivity Analysis

Similar patterns of association were observed with epilepsy and stroke when considering risk of incident dementia at 10 years after the baseline assessment and a follow-up period from 5 to 14 years, which was performed to mitigate potential for reverse causation or undetected dementia at baseline (eTable 7 in Supplement 1). There were 3804 cases of dementia identified for the 10-year follow-up period after baseline assessment and 5110 cases identified for the 5- to 14-year follow-up period. To examine modifiable risk factors more closely, we considered a cardiovascular risk score that did not include genetic *APOE* e4 genotype but instead controlled for the *APOE* e4 genetic risk status in the model. The magnitude of association between each condition and dementia risk was attenuated, but with a similar overall pattern, as participants with high cardiovascular risk and epilepsy had an HR of 7.53 (95% CI, 5.62-10.10) compared with the baseline group of low cardiovascular risk and no epilepsy (eFigure 3 in Supplement 1).

### Epilepsy Subanalyses

Considered an index of epilepsy disease severity, taking more antiseizure medications was associated with lower executive function in those with focal epilepsy (eFigure 4 in Supplement 1). To control for the possibility that the medications themselves might be affecting cognition, we also examined individuals taking antiseizure medications who did not have a diagnosis of epilepsy. In this group, the association was not present (evidenced by a significant interaction between having a diagnosis of focal epilepsy and number of antiseizure medications, *t* = 2.19, *P* = .03). The median age at focal epilepsy onset was 25.5 years, which indicated that seizure onset in this epilepsy cohort generally started during adulthood. Despite the use of more antiseizure medications being associated with a lower executive function, using more antiseizure medications was not associated with a higher risk of developing dementia (eTables 8 and 9 in Supplement 1). The risk of developing dementia associated with late-onset focal epilepsy (age ≥50 years) was comparable (HR, 2.82; 95% CI, 1.97-4.04) with the risk associated with early-onset focal epilepsy (age <50 years: HR, 2.46; 95% CI, 1.93-3.13) (eTable 10 in Supplement 1).

### Brain Structure Analysis

**Figure 4.**
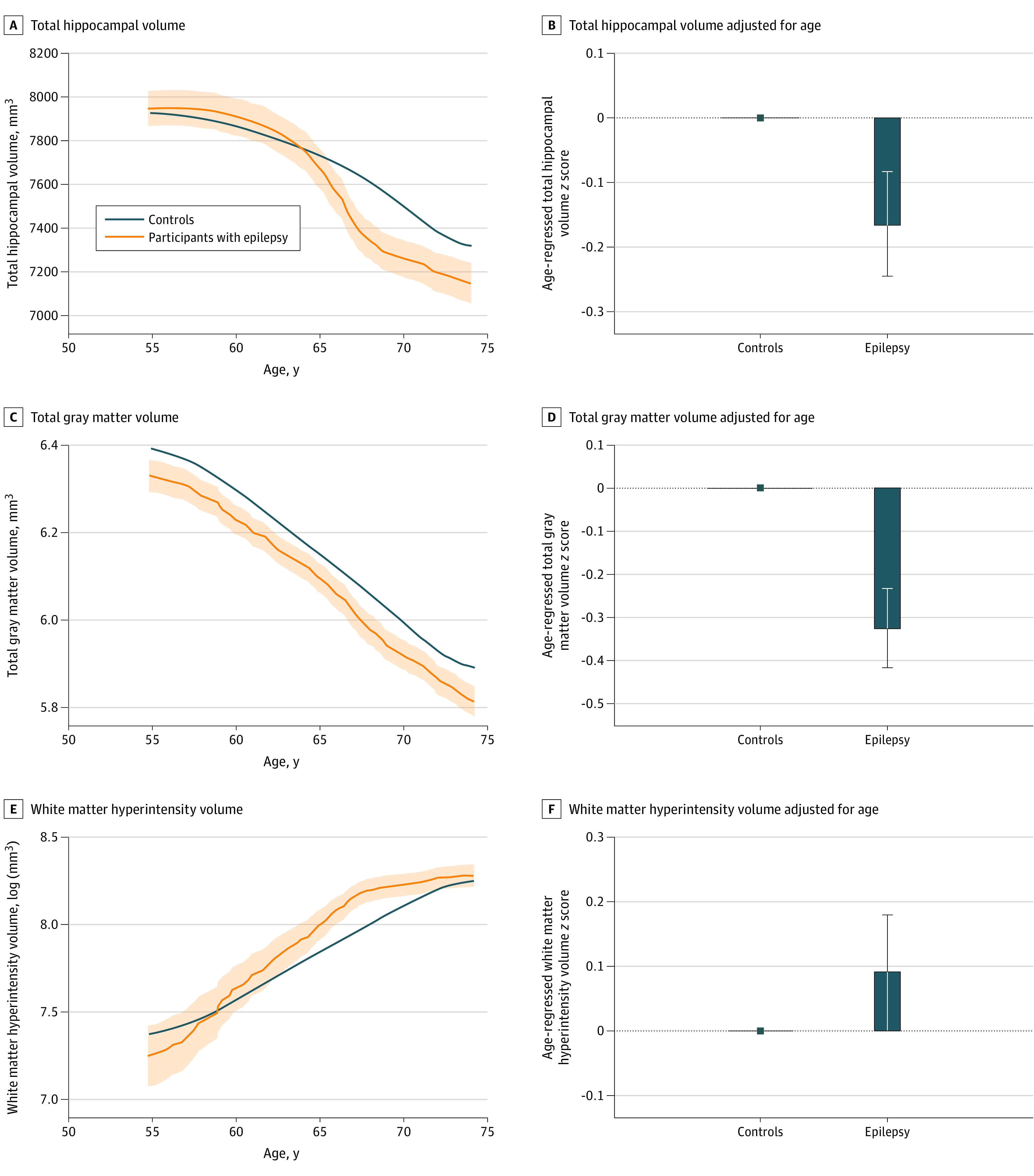
Total Hippocampal Volume, Total Gray Matter Volume, and White Matter Hyperintensity Volume in Participants With Focal Epilepsy and Control Participants Total hippocampal volume was lower in older individuals with focal epilepsy while total gray matter volume was lower in individuals with focal epilepsy of all ages. When regressing out the effect of age, having epilepsy was significantly associated with lower total hippocampal (mean difference, −0.17; 95% CI, −0.02 to −0.32; *t* = −2.18; *P* = .03) and gray matter volume (mean difference, −0.33; 95% CI, −0.18 to −0.48; *t* = −4.29; *P* < .001). No significant difference in white matter hyperintensity was found between individuals with epilepsy and controls (mean difference, 0.10; 95% CI, −0.07 to 0.26; *t* = 1.14; *P* = .26).

Focal epilepsy was associated with lower hippocampal volume (mean difference, −0.17; 95% CI, −0.02 to −0.32; *t* = −2.18; *P* = .03) and lower total gray matter volume (mean difference, −0.33; 95% CI, −0.18 to −0.48; *t* = −4.29; *P* < .001) compared with controls (Figure 4). However, there was no significant difference in white matter hyperintensity volume (mean difference, 0.10; 95% CI, −0.07 to 0.26; *t* = 1.14; *P* = .26). Across age, the difference in hippocampal volume was more apparent in older individuals with focal epilepsy. A fully adjusted linear regression model accounting for other baseline characteristics and cardiovascular risk demonstrated the same pattern of associations (eTable 11 in Supplement 1).

## Discussion

This study showed that having focal epilepsy was associated with worse cognitive function in mid- to late-life individuals compared with controls. Furthermore, by leveraging a large epilepsy cohort with longitudinal data on dementia outcomes, we showed a higher dementia risk in individuals with focal epilepsy compared with those with stroke, which was substantially worse in those with greater cardiovascular risk burden. Focal epilepsy was associated with widespread structural brain change reflected by lower total hippocampal and total gray matter volume.

Several studies have demonstrated worse cognition in epilepsy as individuals grow older,^15,17,35,36,37^ although these are mostly cross-sectional and have generally been small in sample size with fewer than a hundred individuals. One study^38^ showed objective executive function impairment in a larger epilepsy group (n = 257) prior to starting antiseizure medication. With a sample size an order of magnitude larger than previous studies, our current investigation identified worse cognitive function throughout mid- to late-life individuals with focal epilepsy compared with healthy controls, which was comparable with individuals who had a history of stroke. Taking more antiseizure agents was associated with worse cognition in those with focal epilepsy, which may reflect severity of disease in addition to potential for medication adverse effects. Our findings offer an important contribution to understanding the cognitive impact of epilepsy at a group level.

Few studies have suggested epilepsy as a risk factor for dementia^39,40,41,42,43,44^ while other investigations did not find an association.^45,46,47^ Stefanidou et al^48^ identified an increased dementia risk among 43 people with epilepsy in the Framingham Heart study (HR, 1.9; 95% CI, 1.11-3.57) compared with controls, which was slightly lower than our findings. In such studies, cardiovascular risk factors may be controlled for in analyses models or not considered at all. To investigate the potential effect of cardiovascular burden, we stratified individuals based on a previously published cardiovascular risk score^22^ and found more than a 13-fold increased risk of dementia in individuals who have high cardiovascular risk and epilepsy compared with those with no epilepsy and low cardiovascular risk. This increased dementia risk was greater than that of stroke. The lack of association with number of prescribed antiseizure medications excludes an important potential confounder because some older antiseizure medications are also associated with increased vascular risk markers.^49^ Dementia risk associated with early-onset and late-onset epilepsy was comparable in our study, which is consistent with other investigations showing that measures of epilepsy disease duration did not correspond with worse cognitive impairment^50^ or tau pathology burden.^6^ The association between epilepsy and dementia may represent shared risk factors between epilepsy and a vascular dementia-like process^51^ or an interplay between mixed underlying pathology, which is increasingly reported in dementia.^52,53^ In either event, our findings highlight a key clinical message that cardiovascular risk factor modification may be critical for managing cognitive outcomes in focal epilepsy.

Epilepsy neuroimaging studies have observed widespread structural changes in addition to hippocampal atrophy.^28,29,30^ The present study confirms this finding while incorporating important covariates such as education, socioeconomic status, and cardiovascular risk factors, which are often not considered. Structural changes at the whole brain level may reflect widespread network effects of epilepsy regardless of the focal onset of seizures.^54,55^ We did not find a statistically significant difference in white matter hyperintensity burden between individuals with epilepsy and controls, although this trend was present. The finding of widespread structural changes in this cohort is important to understand the effects of epilepsy on the brain.

### Limitations

Our study has several limitations. Because of the observational nature of this cohort, the association of greater incident dementia in epilepsy cannot be taken as causal. Medical information was based on hospital records, death certificate data, or self-report, which may be incorrect.^56^ We identified individuals with nongenetic or focal-onset epilepsy through medical coding; however, this may be inaccurate, and we do not have information on seizure origin, laterality, frequency, or presence of hippocampal sclerosis, which may have an impact on cognitive performance. Such epilepsy characteristics were not captured by the UK Biobank, which was designed to recruit healthy individuals with no single disease in focus. Results of specific clinical investigations, such as electroencephalograms, were not available but would be a beneficial addition to future data releases of the study.

Data from all participants in the UK Biobank, aged 38 to 72 years, were used to include as many diagnoses of epilepsy, stroke, and migraine as possible. We recognize that the younger participants may have a higher chance of developing dementia in later years past the follow-up period of this study. Despite adjustment for potential confounders, the relatively long follow-up period and additional analysis of dementia incidence from 5 to 14 years, there may be unmeasured confounders and potential for reverse causality. While we considered all-cause dementia risk in our study, examining the relationship with dementia subtypes would be interesting; however, subtypes are currently poorly captured in the UK Biobank, and dementia cases are likely to be Alzheimer disease, vascular, or a mixed picture.^57^

The UK Biobank cohort is generally considered healthy and likely to be from less deprived areas^58^; therefore, the effects of cardiovascular risk may be greater in a more representative cohort. Similarly, individuals with focal epilepsy from this cohort are less likely to have drug-resistant, uncontrolled epilepsy that may result in worse cognitive outcomes.

## Conclusions

In this study, focal epilepsy was significantly associated with worse cognitive performance, higher incident dementia risk, and widespread brain differences. Cardiovascular risk was associated with a substantially increased risk of dementia in people with focal epilepsy. Interventions targeting modifiable risk factors may offer an effective management strategy in preventing dementia in individuals with epilepsy.

## Notes

Supplement 1. **eTable 1**. Codes used in the UK Biobank study to identify dementia and cardiometabolic condition cases and exclusion diagnoses **eTable 2. **Codes for anti-epileptic medication recorded in the UK Biobank and simplified medication name **eFigure 1.** Study flowchart **eFigure 2. **Confirmatory factor analysis of computer-based cognitive tasks **eTable 3. **Cardiovascular risk score breakdown **eTable 4.** Post-hoc comparisons examining the differences between individual neurological conditions (mutually exclusive groups) and controls **eTable 5.** Association between Executive Function with neurological conditions using a general linear model controlling for other baseline characteristics **eTable 6.** Dementia risk for focal epilepsy and stroke within each cardiovascular risk group. **eTable 7. **Dementia risk for each neurological condition (mutually exclusive groups) with different follow-up periods **eFigure 3.** Risk of incident dementia associated with focal epilepsy and stroke according to cardiovascular risk groups (calculated without considering APOE e4 status) **eFigure 4.** Relationship between Executive Function and anti-epileptic medication (number and type) **eTable 8. **Association between Executive Function and epilepsy-related characteristics within epilepsy sub-group using a general linear model controlling for other baseline characteristics **eTable 9. **Dementia risk associated with epilepsy-related factors within the sub-group of participants with epilepsy **eTable 10. **Dementia risk for individuals with early- vs late-onset diagnosis of focal epilepsy **eTable 11**. Associations between total hippocampal, total grey matter, total white matter hyperintensity volume with neurological conditions of focal epilepsy, stroke and migraine considering other baseline characteristics.

Supplement 2. Data sharing statement
