# Supplementary material for: Association of Dementia Risk With Focal Epilepsy and Modifiable Cardiovascular Risk Factors
Source: JAMA Neurol. 2023 Mar 27;80(5):445–54. doi: 10.1001/jamaneurol.2023.0339 (PMC10043806; doi:10.1001/jamaneurol.2023.0339)
Supplement: Supplement 1. — eTable 1. Codes used in the UK Biobank study to identify dementia and cardiometabolic condition cases and exclusion diagnoses eTable 2. Codes for anti-epileptic medication recorded in the UK Biobank and simplified medication name eFigure 1. Study flowchart eFigure 2. Confirmatory factor analysis of computer-based cognitive tasks eTable 3. Cardiovascular risk score breakdown eTable 4. Post-hoc comparisons examining the differences between individual neurological conditions (mutually exclusive groups) and controls eTable 5. Association between Executive Function with neurological conditions using a general linear model controlling for other baseline characteristics eTable 6. Dementia risk for focal epilepsy and stroke within each cardiovascular risk group. eTable 7. Dementia risk for each neurological condition (mutually exclusive groups) with different follow-up periods eFigure 3. Risk of incident dementia associated with focal epilepsy and stroke according to cardiovascular risk groups (calculated without considering APOE e4 status) eFigure 4. Relationship between Executive Function and anti-epileptic medication (number and type) eTable 8. Association between Executive Function and epilepsy-related characteristics within epilepsy sub-group using a general linear model controlling for other baseline characteristics eTable 9. Dementia risk associated with epilepsy-related factors within the sub-group of participants with epilepsy eTable 10. Dementia risk for individuals with early- vs late-onset diagnosis of focal epilepsy eTable 11. Associations between total hippocampal, total grey matter, total white matter hyperintensity volume with neurological conditions of focal epilepsy, stroke and migraine considering other baseline characteristics. [file jamaneurol-e230339-s001.pdf]

Supplemental Online Content

Tai XY, Torzillo E, Lyall DM, Sen A, Manohar S, Husain M. Association of dementia risk with focal epilepsy and modifiable cardiovascular risk factors. *JAMA Neurol*. Published online March 27, 2023. doi:10.1001/jamaneurol.2023.0339

eTable 1. Codes used in the UK Biobank study to identify dementia and cardiometabolic condition cases and exclusion diagnoses

eTable 2. Codes for anti-epileptic medication recorded in the UK Biobank and simplified medication name

eFigure 1. Study flowchart

eFigure 2. Confirmatory factor analysis of computer-based cognitive tasks

eTable 3. Cardiovascular risk score breakdown

eTable 4. Post-hoc comparisons examining the differences between individual neurological conditions (mutually exclusive groups) and controls

eTable 5. Association between Executive Function with neurological conditions using a general linear model controlling for other baseline characteristics

eTable 6. Dementia risk for focal epilepsy and stroke within each cardiovascular risk group.

eTable 7. Dementia risk for each neurological condition (mutually exclusive groups) with different follow-up periods

eFigure 3. Risk of incident dementia associated with focal epilepsy and stroke according to cardiovascular risk groups (calculated without considering APOE e4 status)

eFigure 4. Relationship between Executive Function and anti-epileptic medication (number and type)

eTable 8. Association between Executive Function and epilepsy-related characteristics within epilepsy sub-group using a general linear model controlling for other baseline characteristics

eTable 9. Dementia risk associated with epilepsy-related factors within the sub-group of participants with epilepsy

eTable 10. Dementia risk for individuals with early- vs late-onset diagnosis of focal epilepsy

eTable 11. Associations between total hippocampal, total grey matter, total white matter hyperintensity volume with neurological conditions of focal epilepsy, stroke and migraine considering other baseline characteristics.

This supplemental material has been provided by the authors to give readers additional information about their work.

© 2023 Tai XY et al. *JAMA Neurology.*

eTable 1. Codes used in the UK Biobank study to identify dementia and cardiometabolic condition cases and exclusion diagnoses

	Algorithmically-derived	Self-report (includes non-cancer and treatment self report)	Illness code: ICD-10	Illness code: ICD-9
**Dementia**	-	1263	AD: F00, F00.0, F00.1, F00.2, F00.9, G30, G30.0, G30.1, G30.8, G30.9 VaD: F01, F01.0, F01.1, F01.2, F01.3, F01.8, F01.9, I67.3 FTD: F02.0, G31.0, Other codes for all-cause dementia: A81.0, F02, F02.1, F02.2, F02.3, F02.4, F02.8, F03, F05.1, F10.6, G31.1, G31.8	AD: 331.0 VaD: 290.4 FTD: 331.1 Other codes for all-cause dementia: 290.2, 290.3, 291.2, 294.1, 331.2, 331.5
**Stroke**	All-cause stroke: 42006	1081	I630, I631, I632, I633, I634, I635, I636, I638, I639,	43491
**Epilepsy**	-	1264	G40, G400, G401, G402, G405, G406, G407, G408, G409, G41, G410, G411, G412, G418,G419	34540, 34540, 34541, 34550, 34551
**Migraine**		1265	G43, G430, G431, G432, G433, G438, G439	34690
**Diabetes**	-	2443, 6153, 6177,	E10, E100, E101, E102, E103, E104, E105, E106, E107, E108, E109 E11, E110, E111, E112, E113, E114, E115, E116, E117, E118, E119, E12, E120, E121, E122, E123, E124, E125, E126, E127, E128, E129, E13, E130, E131, E132, E133, E134, E135, E136, E137, E138, E139, E14, E140, E141, E142, E143, E144, E145, E146, E147, E148, E149,E15, E150, E151, E152, E153, E154, E155, E156, E157, E158, E159	'25000', '25001', '25009', '25010', '25011', '25019', '25029', '2503', '2504', '2505', '25099'
**Infection of the central nervous system**	-	1244	-	-
**Encephalitis**	-	1246	-	-
**Meningitis**	-	1247	-	-
	-	1262		
**Amyotrophic lateral sclerosis**	-	1259	-	-

© 2023 Tai XY et al. *JAMA Neurology.*

**Multiple Sclerosis**	-	1261	-	-
**Head Injury**	-	1266	-	-
**Subdural Haematoma**	-	1083	-	-
**Subarachnoid Haemorrhage**	-	1086	-	-

© 2023 Tai XY et al. *JAMA Neurology.*

eTable 2. Codes for anti-epileptic medication recorded in the UK Biobank and simplified medication name

Coding	Medication reference	Medication (simplified)
1140856568	acetazolamide [ep] 250mg tablets	acetazolamide
1140875868	acetazolamide	acetazolamide
1140875870	diamox 250mg tablet	acetazolamide
2038459704	carbamazepine	carbamazepine
1140872064	carbamazepine product	carbamazepine
1140872072	tegretol 100mg tablet	carbamazepine
1140863268	clobazam	clobazam
1140863272	frisium 10mg capsule	clobazam
1140872150	clonazepam	clonazepam
1140872152	rivotril 500mcg tablet	clonazepam
1140863152	diazepam	diazepam
1140863170	diazemuls 10mg/2ml injection	diazepam
1140856506	stesolid 5mg/2.5ml rectal solution	diazepam
1140863172	dialar 2mg/5ml syrup	diazepam
1140872160	ethosuximide	ethosuximide
1140872162	emeside 250mg capsule	ethosuximide
1140872164	zarontin 250mg capsule	ethosuximide
1140879744	fenfluramine	fenfluramine
1140867982	ponderax 60mg m/r capsule	fenfluramine
1140872228	gabapentin	gabapentin
1140872236	neurontin 100mg capsule	gabapentin
1140872290	lamotrigine	lamotrigine
1140872302	lamictal 25mg tablet	lamotrigine
1141171932	levetiracetam	levetiracetam
1141171940	keppra 250mg tablet	levetiracetam
1140863302	lorazepam	lorazepam
1140863364	ativan 1mg tablet	lorazepam
1141175204	oxcarbazepine	oxcarbazepine
1141175212	trileptal 150 tablet	oxcarbazepine
2038460068	phenobarbitone	phenobarbital
1140872172	methylphenobarbitone	phenobarbital
1140872174	prominal 30mg tablet	phenobarbital
1140872180	methylphenobarbitone 30mg tablet	phenobarbital
1140872186	phenobarbitone product	phenobarbital
1140850874	cantil+phenobarbitone tablet	phenobarbital
1140909812	methylphenobarbital	phenobarbital
1140910706	phenobarbital	phenobarbital
1141181616	phenobarbital product	phenobarbital
2038460076	phenytoin	phenytoin
1140872098	phenytoin product	phenytoin

© 2023 Tai XY et al. *JAMA Neurology.*

1140872112	epanutin 25mg capsule	phenytoin
1140872304	piracetam	piracetam
1140872306	nootropil 800mg tablet	piracetam
1141200004	pregabalin	pregabalin
1141200072	lyrica 25mg capsule	pregabalin
1140872132	primidone	primidone
1140872134	mysoline 250mg tablet	primidone
1140872198	sodium valproate	sodium valproate
1140872200	epilim 100mg crushable tablet	sodium valproate
1140872214	valproic acid	sodium valproate
1140872216	convulex 150mg e/c capsule	sodium valproate
1140872268	orlept 200mg e/c tablet	sodium valproate
1141172838	depakote 250mg e/c tablet	sodium valproate
1141182592	epival cr 300mg m/r tablet	sodium valproate
1141168436	tiagabine	tiagabine
1141168444	gabitril 5mg tablet	tiagabine
1140923484	topiramate	topiramate
1140927692	topamax 25mg tablet	topiramate
2018602634	vigabatrin	vigabatrin
1140872280	vigabatrin product	vigabatrin
1140872284	sabril 500mg tablet	vigabatrin

© 2023 Tai XY et al. *JAMA Neurology.*

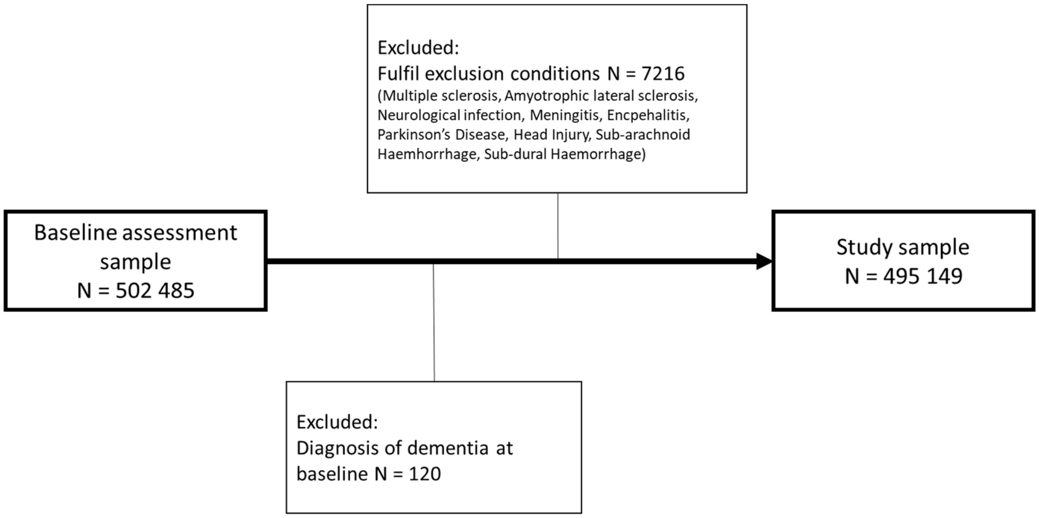

eFigure 1. Study flowchart

© 2023 Tai XY et al. *JAMA Neurology.*

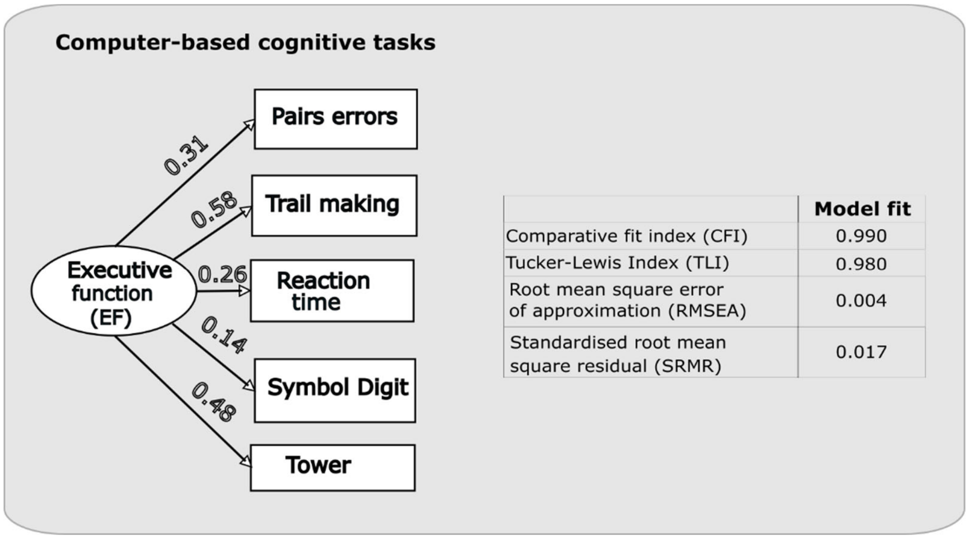

	Model fit
Comparative fit index (CFI)	0.990
Tucker-Lewis Index (TLI)	0.980
Root mean square error of approximation (RMSEA)	0.004
Standardised root mean square residual (SRMR)	0.017

eFigure 2. Confirmatory factor analysis of computer-based cognitive tasks

Path diagram (left) used to create a single latent variable of Executive Function (EF) using confirmatory factor analysis. Fit indices shown on the right.

© 2023 Tai XY et al. *JAMA Neurology.*

eTable 3. Cardiovascular risk score breakdown

	N (%)
**Cardiovascular risk score**	
0	**134249 (27.1)**
1	**169266 (34.2)**
2	**104832 (21.2)**
3	**53932 (10.9)**
4	**24409 (4.9)**
5	**7292 (1.5)**
6	**1109 (0.2)**
7	**60 (0.01)**
**Score components**	
Hypertension	**135882 (27.4)**
High Cholesterol	**81240 (16.1)**
Diabetes	**26945 (5.4)**
WHR (above threshold)	**241931 (48.8)**
APOE one E4 allele	**123636 (24.9)**
APOE two E4 alleles	**11256 (2.3)**

© 2023 Tai XY et al. *JAMA Neurology.*

eTable 4. Post-hoc comparisons examining the differences between individual neurological conditions (mutually exclusive groups) and controls

	Executive Function		
	F	p	
**Neurological conditions & control groups**	201.965	*< .001	
**Post-hoc analysis**	**Mean diff (95% CI)**	**t-stat**	**P_{Tukey}**
**Individual groups**			
Controls – Epilepsy	0.085 (0.070 - 0.099)	14.70	*< .001
Controls – Stroke	0.089 (0.078 - 0.101)	19.89	*< .001
Controls – Migraine	0.002 (-0.006 - 0.010)	0.68	0.906
Epilepsy – Stroke	0.005 (-0.014 - 0.023)	0.67	0.909
Epilepsy – Migraine	-0.082 (-0.099 - -0.066)	-12.82	*< .001
Stroke – Migraine	-0.087 (-0.101 - -0.074)	-16.35	*< .001

Post-hoc analysis of relationship between individual groups of neurological conditions and controls with a Tukey correction applied to account for multiple comparisons. CI- confidence interval.

© 2023 Tai XY et al. *JAMA Neurology.*

eTable 5. Association between Executive Function with neurological conditions using a general linear model controlling for other baseline characteristics

	Executive Function					
	Full Model[a]			Partial Model[b]		
	**β**	**t-stat**	**p**	**β**	**t-stat**	**P**
**Epilepsy**	-0.072	-10.88	**$*< .001$**	-0.081	-14.16	**$*< .001$**
**Stroke**	-0.077	-13.98	**$*< .001$**	-0.063	-19.377	**$*< .001$**
**Migraine**	0.003	1.08	0.270	-0.002	-0.52	0.605
**Age**	-0.012	-168.08	**$*< .001$**	-0.012	-195.37	**$*< .001$**
**CVS risk group**						
**Low**		Baseline				
**Moderate**	-0.017	-12.72	**$*< .001$**	-	-	-
**High**	-0.034	-18.23	**$*< .001$**	-	-	-

[a]*Full model: General linear model adjusted for baseline characteristics including age, CVS risk group, sex, education, socioeconomic status and assessment centre.* β *= unstandardised betas*

[b]*Partial model: Only age included as a covariate together with neurological conditions*

Note: β *= unstandardised betas. CVS – cardiovascular*

© 2023 Tai XY et al. *JAMA Neurology.*

eTable 6. Dementia risk for focal epilepsy and stroke within each cardiovascular risk group

	Dementia risk								
	Low cardiovascular risk			Moderate cardiovascular risk			High cardiovascular risk		
**Subgroup**	**No. of dementia cases**	**HR (95% CI)**	***P*-value**	**No. of dementia cases**	**HR (95% CI)**	***P*-value**	**No. of dementia cases**	**HR (95% CI)**	***P-value***
Controls	495	1 [reference]	-	2695	1 [reference]	-	2281	1 [reference]	-
Stroke	12	4.36 (2.45 – 7.74)	***<0.001	93	2.41 (1.97 - 2.98)	***<0.001	206	1.90 (1.65 - 2.20)	***<0.001
Epilepsy	19	4.81 (3.04 - 7.61)	***<0.001	85	4.23 (3.41 – 5.25)	***<0.001	68	3.73 (2.94 - 4.76)	***<0.001

HRs for participants with stroke only and focal *epilepsy only are compared to a baseline reference group of participants with no history of migraine, stroke or epilepsy. The model has been adjusted for age, sex, education, socioeconomic status and assessment centre.*

© 2023 Tai XY et al. *JAMA Neurology.*

eTable 7. Dementia risk for each neurological condition (mutually exclusive groups) with different follow-up periods

Subgroup	Dementia risk at 10 years			Dementia risk between 5 - 14 years		
	**No. of dementia cases**	**Hazard Ratio (95% CI)**	***P*-value**	**No. of dementia cases**	**Hazard Ratio (95% CI)**	***P*-value**
Migraine	74	0.96 (0.76 - 1.21)	0.732	106	0.99 (0.82- 1.20)	0.930
Stroke	215	2.81 (2.44 – 3.23)	***<0.001	255	2.51 (2.21 - 2.85)	***<0.001
Epilepsy	134	4.93 (4.15 - 5.86)	***<0.001	126	3.52 (2.95 – 4.12)	***<0.001

HRs are compared to a baseline reference group of participants with no history of migraine, stroke or focal *epilepsy. The model has been adjusted for age, sex, education, socioeconomic status and assessment centre.*

© 2023 Tai XY et al. *JAMA Neurology.*

Subgroup	Total No of Participants	No. of dementia cases	Hazard Ratio (95% CI)	P Value
Low CVS risk				
No Stroke/Epilepsy	182560	1191	1 [ref]	
Stroke only	685	21	2.86 (1.77 - 4.61)	<0.001***
Epilepsy only	1375	39	4.53 (3.27 - 6.29)	<0.001***
Moderate CVS risk				
No Stroke/Epilepsy	244592	2871	1.22 (1.14- 1.31)	<0.001***
Stroke only	2988	123	2.97 (2.44 - 3.61)	<0.001***
Epilepsy only	2114	93	4.60 (3.66 - 5.79)	<0.001***
High CVS risk				
No Stroke/Epilepsy	56975	1537	1.82 (1.67 - 1.98)	<0.001***
Stroke only	3030	174	3.50 (2.94 - 4.17)	<0.001***
Epilepsy only	517	47	7.21 (5.32 - 9.77)	<0.001***

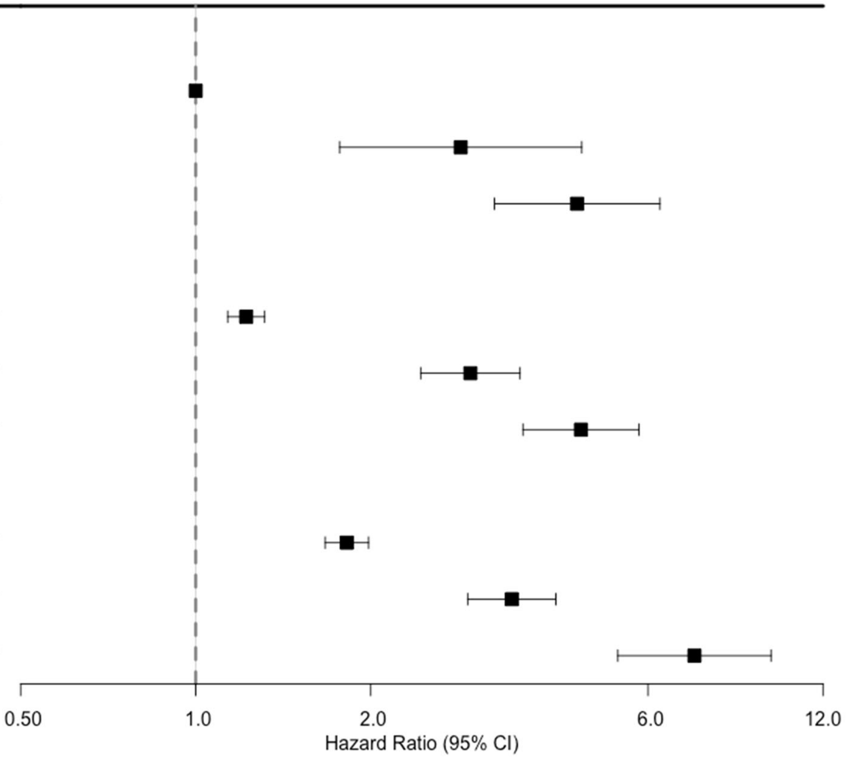

eFigure 3. Risk of incident dementia associated with focal epilepsy and stroke according to cardiovascular risk groups (calculated without considering APOE e4 status)

Data are HRs for incident dementia, associated with groupings of no stroke/epilepsy, stroke only or focal epilepsy only stratified by CVS risk groups (calculated without APOE e4 status). The model has been adjusted for age, APOE e4 genotype, sex, education, socioeconomic status and assessment centre. HR=hazard ratio, CVS = cardiovascular

© 2023 Tai XY et al. *JAMA Neurology.*

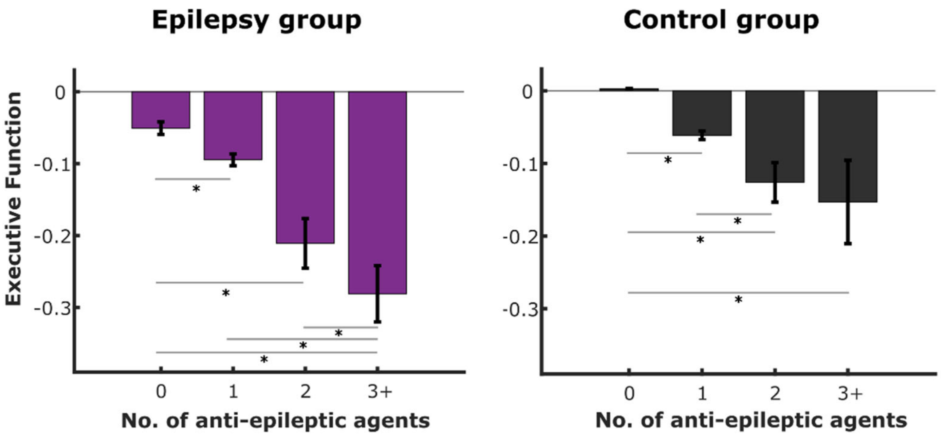

eFigure 4. Relationship between Executive Function and number of anti-epileptic agents in participants with focal epilepsy and controls

Executive Function (z-scored values) is lower in participants with focal epilepsy (left) with increasing number of anti-epileptic drugs. There is a group difference F(3,4225) = 13.61 with post-hoc pairwise comparisons showing a significant difference between taking 0 and 1, 2 or 3 antiepileptic agents (t = 3.43, p_{Tukey} = 0.003, t = 4.29, p_{Tukey} <0.001, t = 5.35, p_{Tukey} <0.001, respectively). There was also a difference between 1 and 3 anti-epileptic agents (3.94, p_{Tukey} < 0.001) and between 2 and 3 anti-epileptic agents (2.72, p_{Tukey} = 0.033). Participants in the control group (right), taking anti-epileptic agents for other reasons, have lower executive function than those who do not take anti-epileptic drugs but to a lesser extent than the focal epilepsy group. There is a group difference F(3,464644) = 65.5 with post-hoc pairwise comparisons showing a significant difference between taking 0 and 1, 2 or 3 antiepileptic agents (t = 11.99, p_{Tukey} < 0.001, t = 6.51, p_{Tukey} <0.001, t = 3.37, p_{Tukey} =0.004 respectively). There was also a significant difference between taking 1 and 2 anti-epileptic agents in this group (t = 3.16, p_{Tukey} = 0.009).

© 2023 Tai XY et al. *JAMA Neurology.*

eTable 8. Association between Executive Function and number of anti-epileptic medication within focal epilepsy sub-group using a general linear model controlling for other baseline characteristics

	Executive Function					
	Full Model[a]			Partial Model[b]		
	β	t-stat	p	β	t-stat	P
**Number of anti-epileptic drugs**	-0.026	-2.20	***0.028**	-0.034	-3.10	**0.002**
**Age**	-0.011	-9.95	***< .001**	-0.011	-11.52	***< .001**

[a]*Full model: General linear model adjusted for baseline characteristics including age, CVS risk group, sex, education, socioeconomic status and assessment centre.* β *= unstandardised betas*

[b]*Partial model: Only age included as a covariate together epilepsy characteristics of disease duration and number of anti-epileptic drugs*

Note: β *= unstandardised betas. CVS – cardiovascular*

© 2023 Tai XY et al. *JAMA Neurology.*

eTable 9. Dementia risk associated with epilepsy-related factors within the sub-group of participants with focal epilepsy

	Risk of incident dementia	
**Epilepsy subgroup**	**Hazard Ratio (95% CI)**	***P*-value**
Number of anti-epileptic drugs	1.17 (0.59 – 1.23)	0.391

This model examines the sub-group of participants with focal *epilepsy. The model has been adjusted for age, sex, education, socioeconomic status, cardiovascular risk group and assessment centre.*

© 2023 Tai XY et al. *JAMA Neurology.*

eTable 10. Dementia risk for individuals with early- vs late-onset diagnosis of epilepsy

Epilepsy subgroup	Dementia Risk	
	Hazard Ratio (95% CI)	*P*-value
Early-onset (<50 yrs)	2.46 (1.93 – 3.13)	***<0.001
Later-onset (>=50 yrs)	2.82 (1.97- 4.04)	***<0.001

HRs for epilepsy only participants are compared to a baseline reference group of participants with no history of migraine, stroke or epilepsy. The model has been adjusted for age, sex, education, socioeconomic status and assessment centre.

© 2023 Tai XY et al. *JAMA Neurology.*

eTable 11. Associations between total hippocampal, total grey matter, total white matter hyperintensity volume with neurological conditions of epilepsy, stroke and migraine considering other baseline characteristics.

	**Total Hippocampal Volume**			**Total Grey Matter Volume**			**Total White Matter Hyperintensity Volume**		
	β	t-stat	p	β	t-stat	p	β	t-stat	p
**Epilepsy**	-112.2	-1.98	***0.047**	-9045.8	-4.46	***< .001**	0.05	0.77	0.444
**CVS risk group**									
Low		Base			Base			Base	
Moderate	-42.6	-5.2	***<0.001**	-2498.4	-8.50	***< .001**	0.06	6.11	***< .001**
High	-117.5	-9.1	***<0.001**	-9033.4	-19.31	***< .001**	0.10	6.14	***< .001**

Note: All volume measures have been deconfounded for age and age^2 at imaging visit, sex, a scaling factor for head size and imaging site. General linear model adjusted for other baseline characteristics including CVS risk group, education and socioeconomic status.

β *= unstandardised betas. CVS – cardiovascular.*

© 2023 Tai XY et al. *JAMA Neurology.*
