# Supplementary material for: Association of Dementia Risk With Focal Epilepsy and Modifiable Cardiovascular Risk Factors
Source: JAMA Neurol. 2023 Mar 27;80(5):445–54. doi: 10.1001/jamaneurol.2023.0339 (PMC10043806; doi:10.1001/jamaneurol.2023.0339)
Supplement: Supplement 2. — Data sharing statement [file jamaneurol-e230339-s002.pdf]

Data Sharing Statement

Tai. Association of Dementia Risk With Focal Epilepsy and Modifiable Cardiovascular Risk Factors. *JAMA Neurol*. Published March 27, 2023. doi:10.1001/jamaneurol.2023.0339

Data
Data available: No

Additional Information
Explanation for why data not available: We will make summary level data and individual field information available but the dataset used in this project (UK Biobank) requires individual researcher data access. This access is widely open to all researchers via an application.
